# Rectification of Material Model for Fibrous Materials in Compressive Mode

**DOI:** 10.3390/ma19071329

**Published:** 2026-03-27

**Authors:** Jūratė Jolanta Petronienė, Rimantas Stonkus, Andrius Dzedzickis, Vytautas Bučinskas

**Affiliations:** Department of Mechatronics, Robotics and Digital Manufacturing, Vilnius Gediminas Technical University, Plytinės g. 25, LT-10105 Vilnius, Lithuania; jurate-jolanta.petroniene@vilniustech.lt (J.J.P.); rimantas.stonkus@vilniustech.lt (R.S.); andrius.dzedzickis@vilniustech.lt (A.D.)

**Keywords:** material model, hyperelastic material model, Yeoh hyperelastic, fibrous material compression, fibrous material stiffness, wool

## Abstract

Fibrous natural-origin materials are not only attractive as raw materials in various applications but are also often produced as waste products in some manufacturing processes. Despite their comprehensive implementation as thermal or noise isolation materials, their behavior under mechanical load is not yet fully understood, and there are no assignments of existing universal material models for the category of fibrous materials. The conducted experimental research provides a methodology with which to evaluate the structural behavior of fibrous materials under applied compression force and classify these materials according to their mechanical properties based on a certain material model. As a result of this research, we observed that the mechanical properties of the fibrous material during compression mode are determined by the fibrous structure, with insignificant influence from the physical nature of the material itself. This investigation provides an analysis of the application of a hyperelastic incompressible isotropic model to fibrous material of different origins. Hyperelastic material models of the Money–Rivlin, Ogden, Yeoh, and polynomial type were implemented. The fitting quality of the Yeoh third-order model obtained the best fitting results for animal wool and mineral wool. Cotton wool showed the best fitting results with the polynomial fifth-order model. The outcome of this research will help create finite element models for structural analysis, efficiently modelling structural responses to vibration or noise. For most animal and mineral wool samples, the best agreement with the experimental compression curves was obtained using the Yeoh third-order hyperelastic model, with coefficients of determination *R*^2^ between 0.979 and 0.996, while fifth-order polynomial fits locally reached *R*^2^ up to 0.9999 for aged cotton wool.

## 1. Introduction

**Figure 1:**
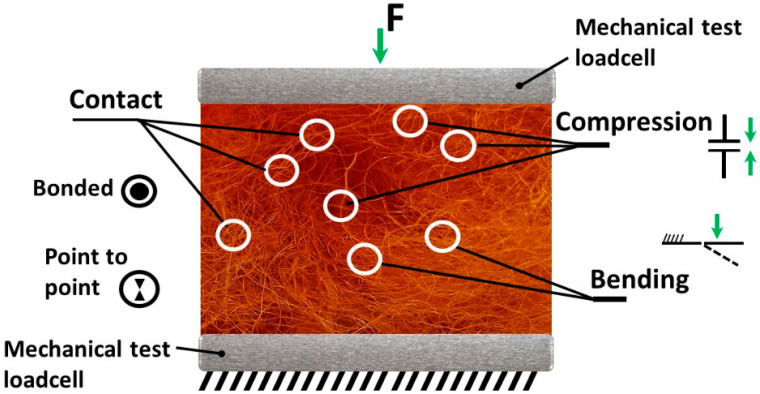
Forces available to influence a single hair during mechanical load.

Recently, the public demand for green building technologies and renewable resources to address the challenges of global warming has become increasingly relevant. Natural-origin materials’ mechanical properties vary naturally over time, reacting to environmental impact by changing the composition of material according to arising challenges. Fibrous materials like wool are a group of materials that acquired a special adaptation to constantly changing natural conditions. Living cells continuously adapt fibrous structures to actual environmental conditions by reorganizing their molecules and structure [1]. Fibrous materials are typically defined as soft materials with features typical of multiphase composition: porous and highly deformable structure, nonlinear elasticity, etc. Their packing structure and geometry are specific and have some similarities with natural-origin biomaterials [2]. Many models of fibrous materials have been developed since the 1930s to predict structural changes and the mechanical behavior of dry fibrous material [3,4]. From that time, the evaluation of multilayer and sophisticated-structure material properties varied with the emergence of new scientific information or new practical needs. Complexity in predicting the mechanical behavior of fibrous material arises due to complex structures containing numerous interaction points between individual fibers (Figure 1), and fiber of organic origin has an even more complex response to mechanical impact, as its properties are influenced by intermolecular interactions [5,6].

The mechanical load that soundproofing products can withstand depends on where and how the material is intended to be used. Although the requirements for acoustic materials are different, they are usually tailored to the installation and real operating conditions for achievable objectives. When planning to use well-known material more effectively or revive abandoned materials, sufficient compressive stiffness is required to prevent structural deformations. The mechanical requirements for the applied materials are already set. In order to achieve economic sustainability and successful application of renewable resources, it is sometimes necessary to find solutions using simulations, such as mathematical models examined using practical experiments.

Wool of various origins is becoming an increasingly popular acoustic material, taking into account fire safety requirements, due to the fact that it is a renewable resource and because of health impact requirements, especially in solving the problems of allergic diseases in humans. Available small deformations of a single-fiber interior sample during the load are presented in Figure 1. The resistance of fiber to applied compression force is the sum of all the processes occurring with the individual fiber and the sum of the frictional forces between them and the elasticity of a single fiber. A macroscopic deformation with a constant value means that the average stress due to the contact and bending of the fiber bundles is constant; the change in the volume fraction occurs only due to the rearrangement of the fiber bundles [7]. In addition to predicting the behavior of fibrous materials, information about the material structure from macroscale to microscale should be considered, since it also impacts the nature and intensity of the interaction between fibers [7,8]. Describing the mechanism of material behavior during mechanical load is a complicated task because weak forces are employed.

Friction between single hairs occurs during mechanical load and can be explained by molecular interaction and the collision of molecules with each other as well as by hysteresis when wool is compressed. Molecular interaction guarantees adhesion on a molecular scale and is responsive to applied force, important for most solids. Friction force increases with applied load, where the normal force is directly proportional to the tangential force through the coefficient of friction. Hence, tangential force is defined as a force that acts on a moving body in interaction or tangent to the curved path of a body [9]. Many efforts were applied to investigate the regularity of fiber bending. The mesoscopic investigation of yarns made by Abbot [10] gave some clarity for predicting yarn quality. However, the thread for products from natural wool is already structured with some regularity. Therefore, when analyzing the behavior of a fibrous material at the microscopic level, it is necessary to take into account the forces of interaction between molecules. For example, van der Waals and electrostatic interactions between molecular structures exist in all materials and play an important role in chain binding [11]. In the case of animal hair, the van der Waals force affects alpha keratin chains’ bonding in wool. Coulomb interactions or salt links are formed between side radicals (R) groups and polypeptide chains when the pH is neutral. These interactions are the result of electrostatic forces of -NH_3_^+^ -COO^−^ groups, and this alpha-keratin property is important for wool’s mechanical properties [12,13]. The mechanical properties of any polymer material depend on molecular chain dynamic abilities and the formed structure [14,15]. Aragon [16] reported that the compression properties of structures containing sisal fiber with higher fiber content resulted in lower compression loads and fiber condition results for polymer matrix elastic and plastic regions in the deformation stress–strain curve. Konopen [17] calculated the elastic constants of the cellulose-containing cell wall. Based on this work, Gassan [18] investigated the anisotropy of cellulose. Plant fiber orientation in the sample determines the response to mechanical effects [19]. Strain ratio parameter ή_l_ allows one to fit the axial strain and the aspect ratio [20]. This strain ratio parameter is used to define the relationship between the fiber axial strain and the aspect ratio [20]. Therefore, by applying model combinations, it is possible to qualitatively evaluate the deformation configurations of a highly stretchable elastomeric nanocomposite. Soft materials can undergo large mechanical deformations prior to final damage under maximum external loads. Due to their multiphase material composition, as usual, hierarchical structure, plastic behavior, and length and time scale effects, they exhibit nonlinearity with associated elasticity, plasticity, and viscosity [21]. Hence, if the material is hyperelastic, then its strain or deformation energy density exists and is a function related to a symmetric strain measure, such that the stress measure is equal to differentiating the strain energy density with respect to the strain measure. Shell structures composed of hyperelastic materials have been widely used in mechanical manufacturing, especially with the development of materials science. In this way, artificial muscles of deformable robots and high-speed micro-actuators are produced. Therefore, the investigation of the nonlinear dynamic behaviors of shells is very important [22]. When deformation is extremely large for the material, the behavior under load shows big differences and depends on the material’s nature, and it is difficult to represent the stress energy density function, which relates the strain and stress. Most of them are biopolymers that exhibit piezoelectric behavior due to the chiral structure of biomolecules, especially cellulose [23]. This and some other facts suggest that chaotic structures where surface phenomena and interaction forces are involved can be described using a mathematical model to provide comfort in simulating the physical properties of new products containing fibrous material. The search for the most suitable model led to a careful investigation of the existing modelling systems.

In general, wool investigators claim that different types of intramolecular bonds exist between polypeptide chains in wool fiber. A study of the mechanical properties of keratin-containing fibers was submitted by M. Feugelman in 1950 [24]. Tensile and bending tests under different loading conditions, defined as stress applied to a given area, are usually performed on raw materials for further use of wool [25]. The 3D visualization of internal damage in the fiber matrix gives practical information. The damage analysis of the structure provides some insights into failure states and enables the prediction of future situations [26]. In addition to the aforementioned data on animal wool, it is necessary to note that this biodegradable and sustainable material addresses environmental and health considerations in building design. The study concludes that sheep wool-based materials can improve room quality and create a more pleasant environment than conventional synthetic materials [27]. Currently, scientists are very interested in animal wool, so additional research is being conducted, and mathematical models are being developed to most effectively use this renewable resource to create hybrid wool and polymer products with increased flame resistance and other applications, thereby improving building structures [28]. The mechanical properties of α-keratin fibers depend on two elongated components of the cortical cells—the highly ordered intermediate α-helix microfibrils and the matrix in which these intermediate filaments are embedded [29]. The structural mechanics still raise important questions regarding crystalline intermediate filaments, amorphous matrices, and the composite mechanics of their interactions [29,30]. Several studies even include single-cell compression, with the ability of individual keratinocytes to withstand external pressure [31]. The contribution of cytoskeletal networks to the observed mechanics of keratinocytes and the unique mechanics of the keratinocyte were described by Lulevich [31]. Thermal insulation materials made with sheep wool as the fiber are highly suitable and durable for thermal insulation [32,33,34]. The Wortmann/Zahn and Feughelman [29] models provide possible qualitative explanations of the stress–strain curve but do not address recovery behavior or explain many other properties, such as cracking, the effect of moisture on mechanical properties, and hardening. Acoustic emission is one of the techniques for investigating the physical properties of fibrous materials, but its accuracy is limited by weak acoustic signals that affect the precision of the investigation [35].

Mineral wool is the most widely used as an insulating material for buildings in northern climates, and, as is usual, it becomes waste after reconstruction [36]. The application of mineral wool in artificial materials is one of the most important ways of promoting sustainability. Good examples of applying mineral wool waste in ceramics, concrete, and asphalt manufacturing exist [37]. Man-made mineral wool has a few types: stone wool, glass wool, and slag wool. Natural stone wool could form under specific natural conditions from effusive rocks. The compression of mineral wool and the resulting irreversible deformation in the articles are considered only as a function of the porosity of the material [38]. Mineral wool, as a form of accumulated waste, has also attracted the attention of scientists due to its potential for reuse [39,40].

In fibrous structure materials, there are a few structural levels: the microstructure, which involves working at the nano-size level forces; the macro-structure, which gives the final design and properties of the material sample; and the meso-structure, the area of interest, which is very pure [41]. Meso-structure is a field of fiber research in which science can improve fiber-reinforced structures more effectively. In realistic fiber-containing compounds, the structure, as usual, has defects, and the investigation of the meso-structure’s influence on the final results of mechanical load is very important. It is especially important for compounds with short fibers. As a meso-structural feature, for example, fiber waviness can be mentioned, which is important for the compressive and shear properties of the composite [42].

The system is considered chaotic if it is three-dimensional and nonlinear [43]. Chaotic systems are common in nature: fluid dynamics, chemical reactions, nonlinear optics, the principles of living tissues, etc. The application of chaos theory is unattractive because the predicted trajectory based on initial conditions and the actual trajectory resulting from the actual initial conditions diverge exponentially until the system differs from the predicted one. A chaotic system has an infinite number of unstable periodic orbits composed into a chaotic set. Two chaotic systems under the same conditions can produce different results.

It is commonly stated that the theory of hyperelasticity was developed for rubber-like structures and should not apply to composite materials such as wool. Daring to defy conventional wisdom, we sought to experimentally demonstrate that, within certain limits, when loads are low, wool can absorb compression and behave as a homogeneous, highly elastic object. This systematic analysis of the approximation of incompressible isotropic hyperelastic constitutive laws to experimental data of fibrous mass of natural origin was conducted, determining material mechanical parameters [44,45,46]. These data were used to check the hypothesis about the material model, which mostly corresponds to general mechanical properties, and to discover a general behavior model, independently of the material origin. The proper material model is suitable for further analysis of structures produced from these materials. Modelling of mechanical behavior can only be ensured by the reliable identification of hyperelastic material parameters. Asadi [47] proposes a method for determining optimal experimental configurations across different deformation ranges, regimes, and hyperelastic models, providing quantitative measurements for experimental design and identifying configurations that reduce noise sensitivity, thereby reducing the number of tests required. Asadi [47] systematized all known theories of hyperelastic materials and developed a simulation to design realistic hyperelastic material characterization experiments, laying the foundation for improved, more efficient material characterization. These insights can be used to expand their applications across biomechanics, engineering, and solid mechanics.

The prediction of chaotically arranged fibrous responses to applied force still requires improvement. Higher-order polynomial models typically provide a good fit to experimentally defined material properties, but they can cause numerical instability when applied to finite element methods [48]. Numerical instability associated with higher-order polynomial approximations is a known problem that can be partially mitigated by applying several standard finite element modeling practices, such as performing mesh convergence analysis, applying incremental loading, and using automatic step control in the nonlinear solver. These measures reduce the risk of solution oscillations and improve the convergence of the numerical procedure. Another solution is to use a constitutive material model that ensures a physically consistent stress–strain relationship over a given strain range.

Furthermore, fibrous materials with partially orientated fibers behave as an anisotropic material, demonstrating different responses to mechanical load concerning the orientation of applied mechanical load concerning the orientation of the applied load with respect to the fiber orientation load with respect to the fibers’ orientation [49,50]. Common elastic materials, under load and after, act according to the model of elastic materials with linear deformation [44]. Therefore, materials are considered elastic when the Piola–Kirchhoff stress law applies to them [51]. P. Xu [52] investigated diffusion models for fiber distribution generation in polymer structures. Lagrangian [53] formulation deals with the nonlinear dynamic equations of isotropic homogeneous hyperelastic materials [54,55]. A nonlinear response to the load comes from the tissues’ microstructural arrangement. A hyperelastic material is frame-indifferent, homogeneous, and isotropic if the stored energy function is equal to a symmetric function of the principal stretches (λ_*x*,*y*,*z*_) [51]. Most soft tissues are described as hyperelastic when they dissipate energy during mechanical load. The dissipation of energy consists of two forms: the Piola–Kirchhoff stress, the function of the deformation F^●^, and Helmholtz free energy, called strain energy [51]. When the system is nonlinear, numerical discretization methods are usually used to compute the total stress by evaluating the directional stress tensor in the unit [56]. The strain energy function describes the stress–strain response during different applied loads [57]. Usually, material models are developed for a large group of materials with similar physical properties. The Rivlin theory of hyperelasticity deals with the stress distribution in an isotropic, incompressible hyperelastic unit cube subjected to three identical pairs of equal and oppositely directed forces *F* of pure homogeneous deformations whose principal directions are parallel to the edges of the cube [58]. The isotropic incompressible solids investigation method was proposed by Mooney and Rivlin [9,10], applying the strain energy function with a linear relationship between shear stress and the amount of shear, between torque and the amount of twist/torsion deformations [59,60]. Based on input parameter values and experimental data, the authors most likely apply the Mooney–Rivlin equation limited by the Gaussian approximation and use the inverse Langevin function form to analyze the three-chain model [29]. Hyperelastic material models include the Mooney–Rivlin [61] model, which uses two principal invariants of the Cauchy–Green strain tensor [62,63,64]. The bending analysis of the defining strain in the cross-section of silicone plates, treating the material as noncompressible and using the Mooney–Rivlin strain energy function, demonstrated the high accuracy of this method [65]. The mechanical behavior of materials can be modelled [66,67,68,69] by focusing only on actual parameters, such as hyperelastic response, and neglecting the material’s viscosity and porosity [70]. Lloyd and Sivaloganathan [71] proved mathematically that Mooney–Rivlin and Ogden material models correspond to the incompressible version for both incompressible and compressible isotropic hyperelastic materials and show that if the unit sphere of an elastic material is subjected to a uniform dead load on the boundary, it is energetically favored to branch into an asymmetric homogeneous deformation rather than radially symmetric cavitated deformation.

K. P. Soldatos [71] studied the states of isotropic, hyperelastic, and compressible expansion of a unit cube, which develops dynamically or reaches and exceeds the cube material’s elastic limit when the instability hinders the deformation. The Cosserat theory [72] of finite deformations of fiber-reinforced elastic solids assumes that the strain energy is an isotropic invariant function of the strain and fiber-spin vector, which differs from the spin vector of the deformation [20]. Continuum theory determines the fiber’s bending stiffness, assuming that the strain energy depends on the strain, fiber direction, and fiber direction gradients in the deformed configuration [73,74]. The generalized equations of stresses and couple stresses are formulated in general and specialized in the case where the dependence on the gradients of the fiber direction is limited to the dependence on their directional derivatives in the direction of the fiber [72]. Linear equations for small strains are derived by Adkins [75] for a sheet containing two sets of cords, and when the cords are initially in parallel lines, these equations are solved in terms of arbitrary functions.

Tiki [76] methods enable the solution of large-scale 3D problems by synchronously optimizing material density and orientation, handling millions of design variables across multiple loading scenarios. Accordingly, the constitutive law for a hyperelastic material relates the strain energy function *Ψ* to the deformation gradient *F*, the invariants, and/or the principal stretches *λ*_1_, *λ*_2_, and *λ*_3_ [22,77]. These molecular dynamics simulations are performed to obtain microscopic responses in hyperelastic materials, especially in compounds with embedded nanomaterials [78]. Mathematical descriptions of hyperelastic material models are also applied to cellular tissues [79,80]. Wegst and Ashby [81,82] presented a comprehensive study of natural-origin materials that show the elastic modulus and strength as functions of density. Mathematical modelling of natural animal wool for predicting moisture sorption is described by Li [83], where thermodynamic variables such as temperature and entropy are held constant. Another important aspect of randomly assembled fibers is the interaction at the point of contact, where fibers can establish an elliptical bonding area proportional to the fiber diameter. At these points, the fibers can bond to each other via cross-linking or adhesion or can remain unbonded. And this is very important for the mathematical modelling of material mechanical behavior. This topological interaction, without cross-linking due to small contact areas from fiber overlap, becomes the main factor in the mechanics of the fiber assembly [80,84]. Adhesive surface interactions come from hydrophobic attraction between filaments, elastic interactions, and hydrogen bonding. Adhesion forces are short range, so special mechanically “activated cellulose” fibers for their stiffness increase when moist. Capillary forces cause elastic fibers to contact via adhesive forces [80]. This is especially significant when studying complex natural fiber structures with infinitely large surface areas, where capillary forces begin to act in response to changes in environmental humidity. The artificial fibrous fabrics exhibit a complex viscoelastic densification response that depends on the architecture and properties of the reinforcement [85,86]. One example of such an effort to describe fibrous materials is Van Wyk’s [87] theory. Ketoja [88] proposed a method to capture complex deformation behavior in a macroscopic mechanical test using Euler’s formula. The contacts that appear during compression have a wide range of contact forces, and only a small part of them do not slip. The predictive ability of this model can be further improved by running larger simulations, changing boundary conditions, and incorporating a more realistic friction model [89]. The Ogden model was established as the most convenient mathematical analysis as an invariant-based model, to introduce a material behavior model under deformation [90] to make a prognosis of the strain–stress behavior of material with no advantage of Rivlin form for rubbers [91], and to describe the nonlinear stress–strain relationship of complex elastomeric materials such as polymers [92]. The polynomial hyperelastic model was proposed by Rivlin and Saunders and is also known as the generalized Rivlin model [93]. The hyperelastic model for prepreg and woven fabric was investigated by D. Sun [42]. The Yeoh hyperelastic model belongs to the family of polynomial models. It is another phenomenological model for the deformation of incompressible materials that agrees well with the third-order Ogden and polynomial models for strain ranging, as stated by several authors [94]. Numerical simulations have been carried out for Neo-Hookean, Mooney–Rivlin, and Ogden models [95] to explore their eligibility/suitability for rubbers within the class of isotropic hyperelastic models. Wen and Hon [96] investigated the nonlinear behavior of the Mindlin plate [97,98,99]. Hyperelastic structures can experience high stresses under external forces. The stress–strain relationship in such structures is complex, so linear stress–strain and linear elasticity models cannot fully capture their mechanical behavior. The Holzapfel–Gasser–Ogden model was reduced to a single scalar quantity describing the mechanical behavior of soft tissues [100,101]. The Neo-Hookean model fits small strains but cannot describe overturn deformation. The sum of the least squares residual error (*Eer*) and the correlation coefficient (*R*^2^) was evaluated to monitor alignment precision [92]. Meanwhile, meshless methods have great power for analyzing nonlinear problems and large deformations because they do not use a mesh grid. Finite element methods (FEMs) are applied to engineering problems, providing solutions by approximating the meshless method constructed in terms of a set of nodes over the computational domain [102,103]. Fiber orientation in the final product, and printing conditions were studied by P. Pibulchinda [103]. Fibrous materials, in some cases, are waste products of the manufacturing process, and provided analysis helps model their sustainable secondary applications [104,105,106]. The special focus on fibrous materials is driven by sustainability considerations [107].

In today’s world, where clean production has gained special importance, it is undoubtedly a useful process that produces no waste and turns environmental waste into a product that is not harmful to health [108]. The successful use of various waste streams in individual regions is determined by locally generated waste that can be used in various compositions in the building materials industry [109].

This investigation contains a literature review on wool structure modeling. Experimental Section 3 presents practical results of mechanical loading on wool-like material structures, including experimental results and fitted curves for different material models. The Discussion of the work is presented in a separate section, while the Conclusion summarizes the data obtained and their potential for planning sustainable solutions for using wool waste in soundproofing systems [104,110,111,112].

Reviewed models of materials correspond to the nonlinear behavior of materials under stress; therefore, based on the experimental outcomes, we use these models—Mooney–Rivlin, Yeoh, Ogden, and polynomial—as they best fit the loading curves. Acceptance criteria for the material model of the researched materials here are assumed to be the quality of approximation by a law-defined curve with fitted parameters. The fitting of experimental results and the fitting outcomes are provided below in the Section 3.

The aim of this work is to define the material model of the fiber material in compressive mode and rectify the model parameters for the chosen materials of organic or mineral origin. These parameters are key to FEM analysis of static and dynamic structural behavior, as well as for the definition of well and contact force. These material properties will enable sound and vibration insulation using FEM tools, which currently lack such capabilities.

## 2. Materials and Methods

### 2.1. Definition of Material Compressive Stiffness Characteristics

**Figure 2:**
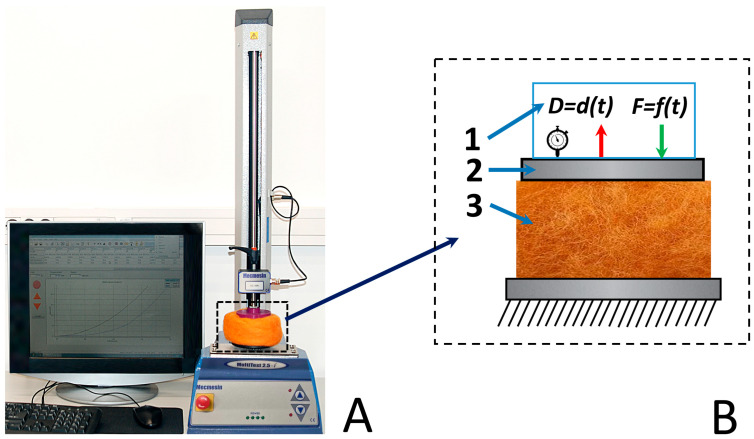
Visual representation of the mechanical test workplace. (**A**) Mechanical compression test system. (**B**) Schematic representation of experimental setup: 1—mechanical force load system; 2—clamps; 3—sample.

Determining the mechanical properties of the investigated fiber material samples was carried out using the computer-controlled tension–compression test system, Mecmesim MultiTest 2.5-i tension compression test system (Mecmesim Ltd., Slinfold, UK, maximum load: 2500 N; maximum sample diameter: 134 mm; load sensor measurement error: ±0.1%; speed range: 1–1000 mm/min). The testing machine was controlled by the software “Emperor” v1.18-408 (Mecmesim Ltd., Slinfold, UK) (Figure 2A), and 25 N and 1000 N load cells were used for the testing machine holders and clamps (Figure 2B).

The experiment procedure is defined as follows:

The sample was placed between pressing plates firmly, but the side peripheries were left open for free displacement of excess material.

The machine runs at an initial speed of 10 mm/min until the load reaches *F* = 0.01 N, with a limited initial approaching distance and a maximum displacement of 20 mm.

The maximum load is limited to 24 N; the loading mode runs with a permanent speed of 10 mm/min until it reaches the force limit.

Release of the sample after compression uses a constant speed of 10 mm/min until it reaches the stroke limit, which is set manually with a slider on the machine.

The other samples were prepared and investigated according to the same procedures.

### 2.2. Sample Preparation

The following materials were tested: cotton, sheep wool, dog wool, and mineral/stone wool. The materials for all experiments were purchased from a local store, except for the dog wool, which was harvested by researchers from pets. The samples of sheep were purchased from the UAB Litwool, (Jonava, Lithuania) shops and from a local sheep breeder. The cotton wool samples were purchased from a pharmacy; the cotton wool was manufactured by Mediteks, Riga, Latvia. The stone wool Paroc (Paroc Group Oy, Helsinki, Finland) was purchased in a local building materials store. All investigated materials are safe to use without a Safety Data Sheet or a CAS number. The samples were prepared for the experiment, maintaining the same cylinder shape and dimensions: diameter *D* = 100 ± 5 mm and height *h* = 50 ± 5 mm for the first series of samples, and the second series of specimens was left with a more naturally formed specimen shape, as a uniaxial type test was performed, and shear was not measured separately. The diameter of the pressing surface is 80 mm. Each sample of each material was prepared from the same unit of testing material to ensure accurate evaluation of the property. All samples were loaded with uniaxial load at a constant speed until a force limit was reached.

Cotton sample. Cotton wool sold in pharmacies is usually in the form of directionally combed fibers. Samples were selected in which the cotton fibers are arranged chaotically. Plant-origin samples selected for experiments were purchased from a pharmacy shop; they were non-sterile and of Turkmen or Uzbek origin. Alternatively, there were samples taken from an aged cotton sample stored for 50 years under moderate conditions: kept at constant room temperature with no sun exposure. Comparison of cotton samples of different ages provides objective data on changes in cotton mechanical properties with age.

Sheep and dog wool. The samples were selected for this study, in which the wool fibers were artificially chaotized from their initial condition to diminish the material’s isotropic behavior. For the test, samples of the following were implemented: (a) raw sheep wool; (b) cleaned layer; and (c) dyed carding product. Samples were taken from Austrian Tyrol-origin sheep wool bream, cleaned, not dyed, with a fiber diameter of 32 µm, and the same manufacturer produced sheep wool bream but dyed. The dog wool was harvested from one of the researchers’ pets, but the dog wool sample was accounted for just for reference to sheep properties evaluation, because a dog’s coat consists mostly of combed undercoat rather than the full-structured coat that results from shearing the animal. Before the experiment, the selected sample was placed in a polyethylene plastic ring to remain on the bench during pressing.

Mineral wool. Samples were selected, including both newly manufactured and aged samples stored for more than 10 years. Only the measurement results for the chaotically arranged fiber samples are presented in this work. Samples were taken from PAROC Ultra mineral wool, with a density of 40 kg/m^3^. In the aged sample, Knauf UNIFIT 035 100 (Knauf Insulation, Shelbyville, TN, USA) was used. For the experiment, an appropriately sized sample was cut and placed on a mechanical stand. All experiments were conducted at 22 °C temperature and 50% humidity. The specimen diameter could vary by up to 10%, while the height was fixed according to the test rig settings, and the loaded area was always defined by the same 80 mm diameter compression plate.

**Figure 3:**
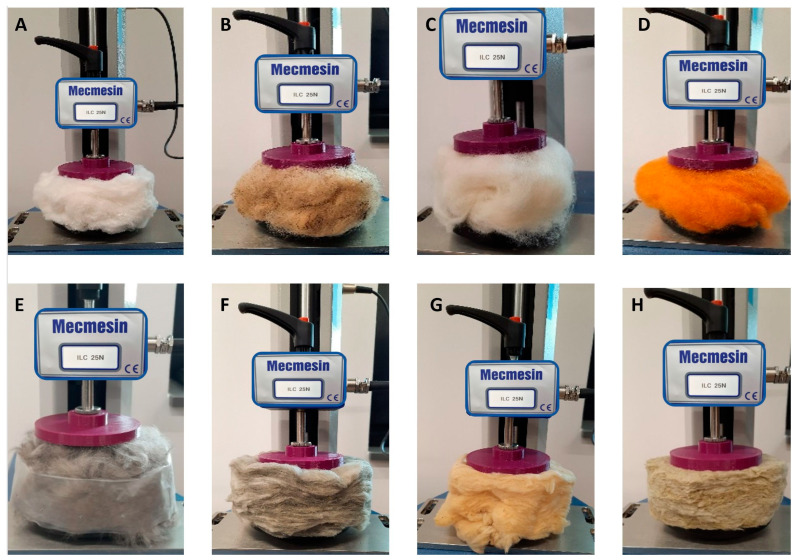
Samples of different materials preserving the natural chaotic shape of the specimen, mounted in a motorized force test stand in the wool stage, were selected for investigation. (**A**) Cotton wool 1; (**B**) sheep wool not cleaned; (**C**) Tyrolen sheep wool bleached; (**D**) Tyrolean sheep wool bleached dyed; (**E**) dog wool; (**F**) mineral wool aged “1”; (**G**) mineral wool “2”; (**H**) mineral wool “3”.

Figure 3 is a visual representation of samples selected to validate the structure’s hyperelasticity, independent of material origin. Samples were prepared to be as natural a shape as they represent after being cut from a larger sample. The cotton wool was stored for over 50 years, packed tightly and isolated from the environment. Other samples of animal origin or sheep wool were as follows: one was taken from a farm just shorn of a sheep, without any cleaning, and the rest were obtained from regular stores after various industrial treatments. Mineral wool was collected in a similar manner. Sample E was taken from a building being demolished, sample G was obtained from a regular hardware store, and sample H was stored openly but was not used in building structures. With this choice, we aimed to assess the impact of the environment, the influence of aging on quality, and the test results.

### 2.3. Refinement of Material Model from Experimental Results

Curve Fitter 2023 v.2.7.8480 software was used to plot and analyze the curves and find the constants of the selected model. The quality of the fit between the experimental stress–strain curves and each hyperelastic model was evaluated using the coefficient of determination *R*^2^, which is reported for all materials and models in the Results section. The experimental data are imported from an MS Excel file, obtained from a measuring machine. The data for the plotted curve were collected from uniaxial compression tests, and the hyperelastic models (Mooney–Rivlin, Yeoh, Ogden) were used to achieve the best concordance with the experimental data.

For the first-order Ogden material model, the strain–energy function (W) for incompressible solid parameters is related as stated in (1):

(1) $$W=\sum_{i=1}^{N}\frac{\mu_{i}}{\alpha_{i}}\left({\bar{\lambda }}_{1}^{\alpha_{i}}+{\bar{\lambda }}_{2}^{\alpha_{i}}+{\bar{\lambda }}_{3}^{\alpha_{i}}-3\right),$$

where *N*, $\mu_{i}$, $\alpha_{1}$ are material constants. Independent variable is engineering strain; dependent variable is engineering stress, with the condition *λ*_1_, *λ*_2_, *λ*_3_* * =  1, which means that the principal stretches *λ_i_* (*i*  =  1, 2, 3) are equal to the modified principal stretches.

The determination was based on a gradually increased extension ratio, defined as *λ*. Reduced stress was calculated from the equilibrium stress of the samples.

Yeoh third-order material model parameters and their relation are given by (2):

(2) $$W=\sum_{i=1}^{N}{C_{i0}\left({\bar{I}}_{1}-3\right)}^{i},$$

where *C*_10_, *C*_20_, *C*_30_ are material constants; they have to be determined through regression analysis. Independent variable is engineering strain; dependent variable is engineering stress. *C*_10_ represents half of the shear modulus of elastic material at the start of deformation, *C*_20_ depends on the degree of softening of elastic material at medium deformations, and *C*_30_ indicates the hardening at large deformation, and they are stiffening constants. ${\bar{I}}_{1}$ is the strain invariant for an incompressible material in tension or compression, according to R.W. Ogden. The relation between stress and stretch for incompressible material under tension or compression is defined by Rivlin.

Mooney–Rivlin three-parameter model is described by (3):

(3) $$W=C_{10}\left({\bar{I}}_{1}-3\right)+C_{01}\left({\bar{I}}_{2}-3\right)+C_{11}\left({\bar{I}}_{1}-3\right)\left({\bar{I}}_{2}-3\right),$$

where $C_{10}$, $C_{01}$, $C_{11}$ are material constants. Independent variable is engineering strain; dependent variable is engineering stress.

Most classical approach of parameter approximation is a fifth-order polynomial relation defined in (4):

(4) $$y\left(x\right)=a+bx+cx^{2}+dx^{3}+ex^{4}+fx^{5},$$

where *a*, *b*, *c*, *d*, *e*, *f* are constants. Independent variable: *x* (engineering strain); depending on variable: *y* (engineering stress).

The experimental results were evaluated for all material models to determine the best-fitting model. The best fit was evaluated using the approximation quality parameter *R*^2^, which is comparable across all materials and implemented approximations. For more complex hyperelastic models, the stress–material Jacobian [47] can be used, and if the Jacobian is material-independent, all material parameters can be expressed as a linear system.

Some investigated materials have fibers stuck together during manufacturing, and groups of fibers arranged in parallel are also observed. All these structural differences, the chaotic nature of the fibers, and the poor ability to predict their arrangement can limit the applicability of a mathematical model [113,114,115].

## 3. Results

After evaluating the information, we assume that the material’s fibrous structure precedes the chemistry of wool’s components. Thus, to provide a hypothesis, we chose plant-origin cotton wool, animal wool, and soft mineral wool samples. Although most of the fibers presented for investigation have a chaotic structure, the investigation was complicated by the fact that individual fibers had some differences in diameter, smoothness, etc.

The hyperelastic material models were revised and adapted for the selected material. For uniaxial extension, the stretch ratio applied and the other two directions can be calculated according to incompressibility. Results of the mechanical load experiment are as follows: curve approximation of the Mooney–Rivlin hyperelastic, Ogden, Yeoh, and polynomial models was performed. In this research, compression strain–stress curves are used to explain regression analysis processes. The average compressive response is usually described quantitatively by a universal curve, in which the relative changes are independent of fiber properties, assuming the fibers are similarly bonded.

**Figure 4:**
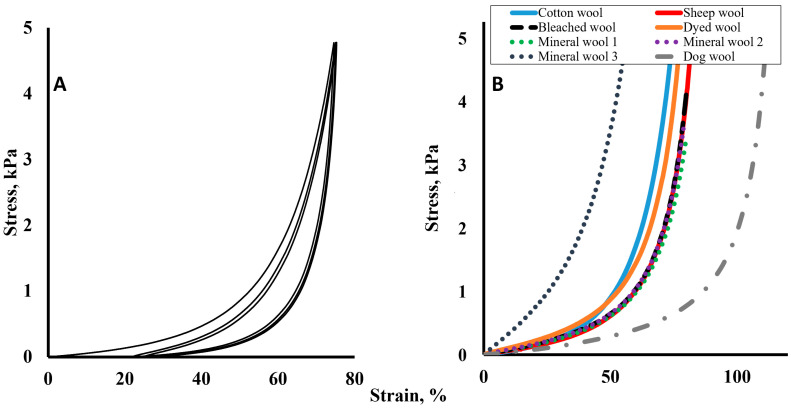
Cotton wool under load deformation curves with approximation curves. (**A**) Stress–strain curve for three cycles of the uniaxial compression test of cotton wool samples; (**B**) stress–strain curve of uniaxial compression test data of cotton wool sample and three material models: uniaxial Mooney–Rivlin three-parameter, uniaxial Ogden first-order and uniaxial Yeoh third-parameter models.

Figure 4A represents the hysteresis of the mechanical load of cotton wool. The hysteresis loops are increased during the first few cycles.

In Figure 4B, the results of plant wool, animal, and mineral wool investigations are represented for comparison. As we can evaluate from Figure 4B, differences in the results of the mechanical load of animal wool are under the influence of keratin “softness” in comparing the grey line of soft dog hair and the red line of sheep hair [115]. Also, the differences between the measurement results for animal-origin wool (dog and sheep) are greater than those for mineral wool and sheep wool. The results of the material model approximation are provided in Table 1.

For aged cotton wool, the fifth-order polynomial model achieved *R*^2^ = 0.9999, the Yeoh third-order model *R*^2^ = 0.9982, and the Ogden first-order model *R*^2^ = 0.9887, whereas the Mooney–Rivlin three-parameter model yielded *R*^2^ = 0.9488.

**Figure 5:**
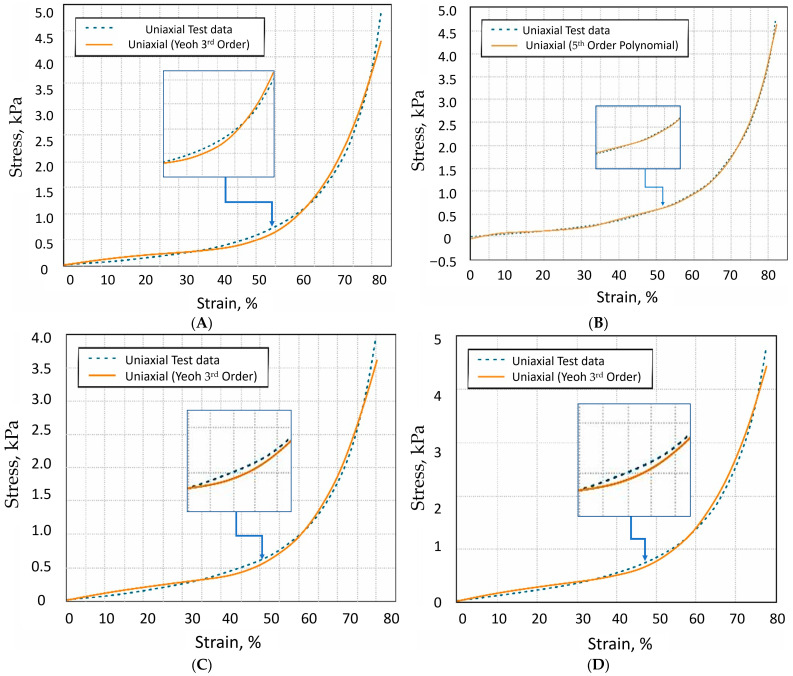
Investigation of animal-origin wool under mechanical load with an applied mathematical model. (**A**) Stress–strain curve of uniaxial compression test data of sheep wool sample and uniaxial Yeoh third-order fit curve (orange line); (**B**) stress–strain curve of uniaxial compression test data of sheep wool sample and uniaxial fifth-order polynomial fit curve (orange line); (**C**) stress–strain curve of uniaxial compression test data of Tyrolean sheep wool (bleached) sample and uniaxial Yeoh third-order fit curve (orange line); (**D**) stress–strain curve of uniaxial compression test data of Tyrolean sheep wool (orange dyed) sample and uniaxial Yeoh third-order fit curve (orange line).

Sheep wool received the most attention in the investigation because it has the widest application in the industry and causes the most problems as waste. For this investigation, we selected wool products with varying levels of industrial processing. We investigated other samples to show how fiber diameter and chemical treatment affected structural variations that reflect the response to mechanical load. Aged natural sheep wool, freshly collected specimen of natural sheep wool, Austrian Tyrol bleached sheep wool for handicraft, and dyed Austrian Tyrol sheep wool were investigated. To evaluate aging processes in the natural keratin product, an investigation of the influence of pressure on the samples was performed under an optical microscope. Obviously, keratin products are resistant to rapid aging when wool samples are stored in a natural environment where decay-causing bacteria cannot act. Figure 5 represents the results of the mechanical load and the mathematical model approximation results of the sheep wool mechanical investigation.

As shown in Figure 5, the experimental data curves are best fit by the polynomial model. It is known that the Ogden third-order model expresses the strain energy at large strains for very flexible materials, but the polynomial fifth-order model expresses the strain tensor invariants in intermediate ranges. Both models are applicable for moderate compression. At high compressions, higher-order models may cause unrealistic predictions. Thus, by applying both models, we can assess the suitability of a material when combined with other materials for creating composite structures and predict the loads a wool-like structure may experience. The inset of the figure shows the maximum deviation between the practical data and the fitted curve. The results obtained do not contradict the statements in scientific articles that, for small deformations, the polynomial-order model is more suitable, while Ogden describes large deformations more accurately. The *R*^2^ of Yeoh’s third-order model is higher than that of the Mooney–Rivlin or other hyperelastic material models. Constants of approximation to mathematical models are represented in Table 2.

Since animal wool also has underwool, composed of much finer hairs, we investigated dog wool as a softer animal fur [116]. Since there are already publications comparing the physical properties of sheep wool and dog wool, this material was chosen for comparison to expand knowledge and applicability models for scientists who design the parameters of wool-containing building materials [117].

**Figure 6:**
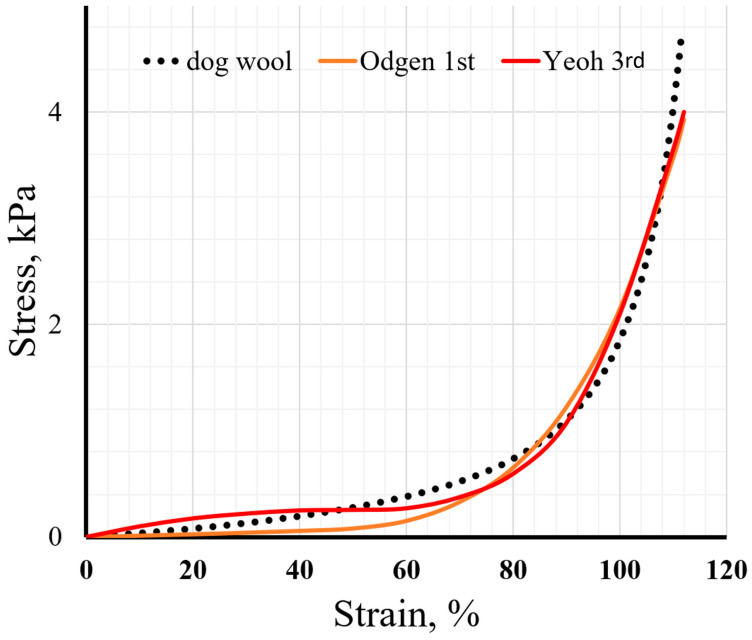
Dog wool mechanical investigation. Orange line: stress–strain curve of uniaxial compression test data of a dog wool sample and uniaxial Ogden first order; red line: stress–strain curve of uniaxial compression test data of a dog wool sample and uniaxial Yeoh third order; black dots: experimental results.

Results of dog wool mechanical load experiments are represented in Figure 6 and in Table 3, respectively.

When wool is soft, it is easy to compress, and it is difficult to apply a hyperelastic material model. For this experiment, wool collected from a dog or other domestic animal can be added to the list of sustainable uses for raw materials, but predicting its behavior would be too complicated due to the diversity of pet breeds and differences in fur care. The Yeoh third-order model is most suitable for describing the behavior of dog wool under mechanical compression.

For sheep wool samples, *R*^2^ values for the Yeoh third-order model ranged from 0.992 to 0.995, while the corresponding fifth-order polynomial fits reached *R*^2^ up to 0.999. In quantitative terms, the best fits of the hyperelastic models to the experimental compression curves yielded coefficients of determination *R*^2^ above 0.98 for most materials, with the fifth-order polynomial model for aged cotton wool reaching *R*^2^ = 0.9999 and the Yeoh third-order model giving *R*^2^ between 0.992 and 0.995 for industrially processed sheep wool.

The identified compressive response of chaotic fibrous structures spans from relatively soft dog wool (*R*^2^ = 0.979 for the Yeoh third-order fit) to very stiff fresh mineral wool (*R*^2^ ≈ 0.9999), demonstrating that the selected hyperelastic models can accurately reproduce the behavior of both organic and mineral fibrous materials over the investigated strain range.

The chaotic arrangement of fibers in mineral origin wool allows for the assumption that the mechanical properties of such a product can be influenced not by the mineral origin nature but by the structure of the chaotic arrangement, when the sample is subjected to mechanical load. It is generally considered that hyperelastic models are not suitable for the compression of mineral wool. Stone or mineral wool acts differently under compression due to its nature. During the compression of the mineral wool, void densification occurs, which is an irreversible form of deformation, and it does not return to its original thickness. The hyperelastic models can be applied for compression measurement only in the initial elastic range, when the mineral wool does not collapse. Finite element material models are used to describe such materials, which are most commonly used to model mineral wool.

**Figure 7:**
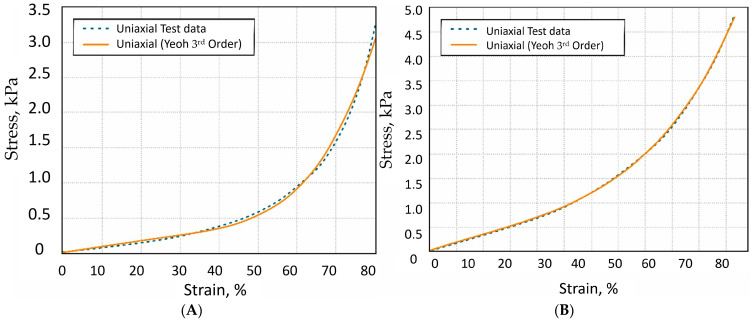
Investigation of mineral wool using mechanical load and approximation of mathematical models of uniaxial Yeoh third order. (**A**) Uniaxial Yeoh third-order fitting for mineral wool 1 (aged); (**B**) uniaxial Yeoh third-order fitting for mineral wool 3 (newly produced).

For this experiment, aged samples were selected to evaluate the potential of such waste. Results of the investigation are represented in Figure 7 and Table 4.

Mineral wool is an artificial structure. The difference in the mineral wool structure compared to other investigated products is that the fibers have permanent connections that form during the melting process. Amorphous mineral structure in nature is more like a liquid than a crystalline structure. Therefore, a crystalline structure responds differently to mechanical load than a “slow-flowing liquid”. The aged samples were stored for a few decades (Figure 6, black dots). They are covered by dust from human activities, and in some cases, the fiber structure, filled with dust, has a certain analogy to other fibers, where the natural-origin structure is arranged in layers, and the upper layer forms a certain flexibility enabled by wax or fat.

## 4. Discussion

**Figure 8:**
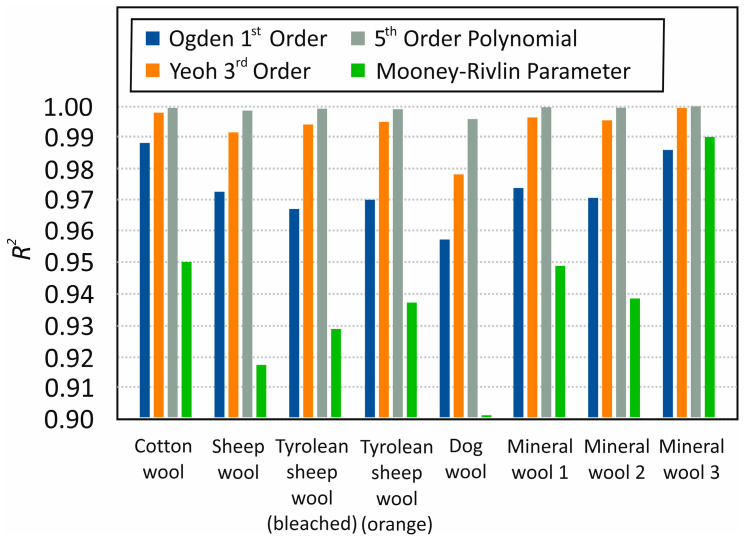
Statistics of the investigation. Comparing the hyperelastic model (Ogden first order, Yeoh third order, polynomial fifth order, Mooney–Rivlin three parameter) application on different fibrous materials: cotton wool, natural sheep wool, bleached sheep wool, dyed sheep wool, dog wool, aged mineral wool, mineral stone wool, mineral glass wool.

In this investigation, the compressive strength model of low-density randomly arranged fiber networks was evaluated, assuming that the bending of fiber segments dominates the strength behavior throughout the compression curve. This study investigated the behavior of fiber blocks placed between two plates under combined compressive loading using experimental and approximate analytical theories. Existing analytical theories for compressive loading were reviewed, and their accuracy was evaluated based on tests. The statistics for the approximation of the results of all investigated materials are presented in Figure 8. Evaluating selected hyperelasticity models to describe material behavior under mechanical compression, we state that the most precisely consistent model for compression effects on fibrous material is the Yeoh third-order model. According to the final results, the fiber’s response to mechanical compression is determined by the material’s fiber structure and its chaotic arrangement. We reached this conclusion by applying various models for hyperelastic materials and obtaining similar results. The finite element update method was applied to material model parameters that could not be measured directly.

As is known, good quantitative predictability is usually lacking when using materials of biological origin, which are characterized by chaotic structures and variability due to changes in environmental conditions. According to the statement, based on experiments by other authors, in the case of non-binding fibers, deviations from the theory are observed only when additional membrane structures form within the fiber network using polymer binders [88]. However, the aforementioned theory applies to porous or fibrous products in which the fibers are physically bonded. Therefore, to fully evaluate fibrous structures, we supplement our knowledge by applying hyperelastic material models. It is likely that a statistical universality similar to that described in this study will hold for many other types of random fiber-optic networks, but a detailed study of other naturally occurring fiber-optic structures may yield greater precision and even a different assessment. To observe quantitative universality more effectively, it is necessary to collect data from a large number of experimental samples, since individual samples of the same material may differ due to surface effects, local density fluctuations, and other features not accounted for by the theory. Whereas animal wool and cotton wool are complex, layered, and chemically heterogeneous, they are naturally formed to withstand various external factors and mechanical compression. Data evaluation is complicated by the way these natural fibers adapt to constantly changing atmospheric conditions.

## 5. Conclusions

Although lumped Ogden models with these exponents are consistent with data from other experiments, the analysis presented here suggests that these models are perhaps too simple to capture the important effects of normal stress in simple shear. Thus, as in most applications of these models, additional terms are needed to form a fully realistic model. The hyperelasticity models Neo-Hookean, Mooney–Rivlin (three- and five-parameter), Ogden (first, second, third terms), Yeoh (first, second, third orders), and Arruda–Boyce were applied to investigate materials of different origins. Across all investigated fibrous materials, the identified hyperelastic models provided a very good description of the compressive response, with *R*^2^ typically above 0.98 and up to 0.9999 for the best-fitting cases. In addition, experimental data from a uniaxial tensile test were evaluated and compared. We found that the most accurate material model simulating both uniaxial tension and shear loading was Yeoh’s third-order material model; however, this accuracy would be valid only over a small range of deformations. All processes for creating deformation models are based on two mechanisms: one governs behavior at small strains, and the other dominates at large strains. Network defects, such as entanglement, are suspected of being responsible for the first mechanism. The second reason is the finite expansion of network circuits. Thus, macroscopic behavior is directly related to molecular concepts. By comparing natural fiber materials and mineral wool, we assess the feasibility of replacing them in the construction sector. To summarize, the results were included in the comparison of hyperelastic models applied to natural fiber-structured materials. In this work, the theoretical prediction proved applicable across the entire range of selected materials studied. It will be interesting to evaluate the validity of the reasoning in the presence of other fibrous structures of organic origin.

- The investigation results show the following: the decisive factor in the material response to the mechanical compression is not the nature of the material (vegetation, animal, mineral origin) but its structure (in this case, chaotically arranged fibers).
- The application of hyperelastic material models to chaotic fiber structures is not only based on considerations about the analogy of the structures compared to confirmed hyperelastic materials.

Natural fibers and wool, as well as mechanically produced mineral wool, can be used in industries that require elastic fillers due to their physical structure. The possibilities of using such materials can be predicted by applying the hyperelastic Ogden and Yeoh models.

- Fibers formed into a cylindrical sample, in this case, responded to compression as hyperelastic materials. The ability to provide detailed information limited the evaluation of how shear deformations work and the influence of fiber orientation on the mechanical samples.
- At the global level, the implementation of waste utilization in the real construction sector is promoted, contributing to the preservation of natural resources and the environment.
- The fibers of living organisms, developed by nature over millions of years, are perfectly adapted to withstand the effects of the atmosphere, and climate challenges have perfectly adapted materials to withstand mechanical loads; you just need to use these materials properly. Sustainability requires more careful use of generated waste. This research and applied mathematical model can serve as a basis for the application of these natural fiber materials in the development of new compositions of materials for different industries.

## Figures and Tables

**Table 1:** Material model approximation results of cotton wool (aged) mechanical compression.

Material Model	Constants	Fitting Quality (*R*^2^)
Ogden 1st order	*µ*_1_ = 4.18345 × 10^−11^; *α*_1_ = 5.26923	0.988663
Yeoh 3rd order	*C*_10_ = 4.54629 × 10^−6^; *C*_20_ = −6.77114 × 10^−10^; *C*_30_ = 3.40742 × 10^−13^	0.99818
Polynomial of 5th order	*a* = −2.812 × 10^−5^; *b* = 1.89828 × 10^−5^; *c* = −1.64778 × 10^−6^; *d* = 7.96767 × 10^−8^; *e* = −1.51922 × 10^−9^; *f* = 1.14632 × 10^−11^	0.999903
Mooney–Rivlin 3 Parameter	*C*_10_ = −2.69902 × 10^−5^; *C*_01_ = 26.1328 × 10^−5^; *C*_11_ = 2.15508 × 10^−7^	0.948827

**Table 2:** Material fit to model results of sheep wool mechanical compression experiments.

Material Model	Wool Sort	Constants	Fitting Quality (*R*^2^)
Uniaxial Yeoh 3rd order	Fresh harvested	*C*_10_ = 5.692; *C*_20_ = −0.00116147; *C*_30_ = 2.52572 × 10^−7^	0.991795
Uniaxial 5th Order Polynomial	Fresh harvested	*a* = −72.0981; *b* = 39.3651; *c* = −3.5033; *d* = 0.140263; *e* = −0.00231877; *f* = 1.41536 × 10^−5^	0.998945
Uniaxial Yeoh 3rd order	Tyrolean sheep wool bleached	*C*_10_ = 5.77596; *C*_20_ = −0.000966824; *C*_30_ = 2.26213 × 10^−7^	0.994163
Uniaxial Yeoh 3rd order	Tyrolean sheep wool dyed	*C*_10_ = 7.11097; *C*_20_ = −0.00113851; *C*_30_ = 3.05313 × 10^−7^	0.995447

**Table 3:** Results of approximation to mathematical model of dog wool compression experiments.

Material Model	Constants	Fitting Quality (*R*^2^)
Uniaxial Ogden 1st order	*µ*_1_ = 1.57546 × 10^−8^; *α*_1_ = 6.55448	0.957496
Uniaxial Yeoh 3rd order	*C*_10_ = 4.7873; *C*_20_ = −66.9977× 10^−5^; *C*_30_ = 6.17974 × 10^−8^	0.978999

**Table 4:** Results of the mathematical modelling of the uniaxial Yeoh third order of mechanical load of mineral wool.

Type of Mineral Wool	Constants	Fitting Quality (*R*^2^)
Aged mineral wool, stored	*C*_10_ = 4.71072; *C*_20_ = −0.000529304; *C*_30_ = 1.63934 × 10^−7^	0.996894
Fresh insulating mineral wool	*C*_10_ = 15.0101; *C*_20_ = 0.00147215; *C*_30_ = 5.1852 × 10^−7^	0.99991

## Data Availability

The original contributions presented in this study are included in the article. Further inquiries can be directed to the corresponding author.
